# A Mass Spectrometry Imaging Based Approach for Prognosis Prediction in UICC Stage I/II Colon Cancer

**DOI:** 10.3390/cancers13215371

**Published:** 2021-10-26

**Authors:** Benedikt Martin, Juliana P. L. Gonçalves, Christine Bollwein, Florian Sommer, Gerhard Schenkirsch, Anne Jacob, Armin Seibert, Wilko Weichert, Bruno Märkl, Kristina Schwamborn

**Affiliations:** 1 Institute of Pathology and Molecular Tumor Diagnostics, University Hospital of Augsburg, 86156 Augsburg, Germany; benediktm@googlemail.com (B.M.); bruno.maerkl@klinikum-augsburg.de (B.M.); 2 Institute of Pathology, School of Medicine, Technical University of Munich, 81675 Munich, Germany; juliana.goncalves@tum.de (J.P.L.G.); christine.bollwein@tum.de (C.B.); anne.jacob@tum.de (A.J.); wilko.weichert@tum.de (W.W.); 3 Department of Visceral Surgery, University Hospital of Augsburg, 86156 Augsburg, Germany; florian.sommer@uk-augsburg.de; 4 Cancer Tumor Data Management, Comprehensive Cancer Center, University Hospital of Augsburg, 86156 Augsburg, Germany; Gerhard.Schenkirsch@uk-augsburg.de; 5 Institute of Mathematics, Augsburg University, 86159 Augsburg, Germany; seibert.armin@googlemail.com

**Keywords:** colon cancer, mass spectrometry imaging, proteomics, MALDI, tumor prognosis

## Abstract

**Simple Summary:**

Tumor treatment is heavily dictated by the tumor progression status. However, in colon cancer, it is difficult to predict disease progression in the early stages. In this study, we have employed a proteomic analysis using matrix-assisted laser desorption/ionization mass spectrometry imaging (MALDI-MSI). MALDI-MSI is a technique that measures the molecular content of (tumor) tissue. We analyzed tumor samples of 276 patients. If the patients developed distant metastasis, they were considered to have a more aggressive tumor type than the patients that did not. In this comparative study, we have developed bioinformatics methods that can predict the tendency of tumor progression and advance a couple of molecules that could be used as prognostic markers of colon cancer. The prediction of tumor progression can help to choose a more adequate treatment for each individual patient.

**Abstract:**

Currently, pathological evaluation of stage I/II colon cancer, following the Union Internationale Contre Le Cancer (UICC) guidelines, is insufficient to identify patients that would benefit from adjuvant treatment. In our study, we analyzed tissue samples from 276 patients with colon cancer utilizing mass spectrometry imaging. Two distinct approaches are herein presented for data processing and analysis. In one approach, four different machine learning algorithms were applied to predict the tendency to develop metastasis, which yielded accuracies over 90% for three of the models. In the other approach, 1007 *m*/*z* features were evaluated with regards to their prognostic capabilities, yielding two *m*/*z* features as promising prognostic markers. One feature was identified as a fragment from collagen (collagen 3A1), hinting that a higher collagen content within the tumor is associated with poorer outcomes. Identification of proteins that reflect changes in the tumor and its microenvironment could give a very much-needed prediction of a patient’s prognosis, and subsequently assist in the choice of a more adequate treatment.

## 1. Introduction

In 2012, about 14.1 million new cancer cases occurred worldwide and approximately 6 million occurred in developed countries [1]. In developed countries, about one of eight was of colorectal origin [1]. Cancer diagnosis is usually based on pathological evaluation, to facilitate an adequate therapeutic approach. Besides diagnosis, prediction of disease progression is the main task of pathological examination. Up till now, the backbone of risk stratification is still the TNM-classification. However, in the Union Internationale Contre Le Cancer (UICC), which stipulates the guidelines for TNM-classification, the definition of stage I/II colon cancer is insufficient for prognosis prediction. The majority of these patients show a favorable clinical course. For this reason, adjuvant chemotherapy is restricted to particular situations (pT4, inadequate lymph node harvest, obstruction, and emergency surgery) in stage II cancer [2]. For UICC stage II colon cancer, the five-year disease-free survival is estimated to be approximately 81% without adjuvant chemotherapy and approximately 79% with adjuvant chemotherapy, respectively [3]. However, up to 20% of patients show a progressive clinical course and could benefit from adjuvant chemotherapy [3,4]. In view of these sobering data, the development of a personalized therapy concept is essential. Therefore, advanced biomarkers for the prediction of the clinical course are required in order to identify patients in need of adjuvant treatment. Promising biomarkers, such as microsatellite status, tumor-infiltrating immune cells, and poorly differentiated clusters have been suggested [5,6,7]. Nevertheless, only tumor budding has been generally recommended in node-negative cases since the publication of the international tumor budding consensus conference [8]. At the same time, some patients do not benefit from adjuvant therapy but suffer from side effects [9,10].

We have investigated a new approach for risk stratification by in-situ proteomic analysis using matrix-assisted laser desorption/ionization mass spectrometry imaging (MALDI-MSI). MALDI-MSI is a powerful, label-free technique, which allows the investigation of biomolecules (such as peptides and proteins) and their spatial distribution within intact tissue sections [11,12,13,14]. With this technology, one mass spectrum is collected, with a predefined laser spot-size/resolution. After spectrum acquisition, the target plate moves to the following x/y position within the tissue to collect a new spectrum. This process is repeated until the whole tissue section or an area of interest is measured. To better illustrate, each tissue microarray (TMA) core of 1 mm in diameter, as used in this study, contains over 500 individual spectra when defining a resolution of 50 µm. For larger studies, formalin-fixed paraffin-embedded (FFPE) samples are commonly used due to easier processing and availability, but the approach can also be extended to fresh-frozen tissue samples [15]. By correlating the mass spectra with the precise position where they were collected it is possible to create distribution maps of all measured molecular ions across the tissue. Moreover, after measurement, it is possible to perform comparative analysis with hematoxylin and eosin (H&E) or immunohistochemistry staining utilizing the very same tissue section, allowing direct correlation with histomorphological characteristics of the tissue [16,17]. While conventional approaches, such as immunohistochemistry and radiochemistry, are limited by tissue availability and/or the number of analytes measured, MSI can record an uncountable number of analytes without the need for target-specific reagents using solely one tissue section. In that direction, methodologies aiming to evaluate different types of analytes (proteins, glycans, lipids, etc.) in the same sample are also being developed [18,19].

In this study, we examined whether a proteomic approach using MALDI-MSI can predict the risk of progression (as defined by occurrence of distant metastases) in patients with UICC stage I/II colorectal cancer.

## 2. Methods

### 2.1. Sample Collection

We performed this study utilizing a patient cohort previously reported [20]. In brief, the cohort was identified by searching the administrative database of the Institute of Pathology and Molecular Diagnostics of the University Hospital of Augsburg to identify all cases of UICC stage I/II colon cancer with a minimal follow-up period of two months. Samples from patients with surgical treatment between 2002 and 2007 were included in the study (patients from 2006 were excluded due to different pathologic preparation techniques). Only patients with negative resection margins and no clinical or pathologic evidence of metastatic disease at the time of diagnosis were included. The patients were treated in accordance with the valid guidelines at the time of treatment and therefore chemotherapy was not provided as the standard treatment. Additional 21 cases had to be excluded due to small tumor size (which could have resulted in unacceptable tissue loss), and four were excluded since the sample was not available. The final cohort was composed of samples from 276 patients.

The follow-up data was provided by the Clinical and Population-based Cancer Registry Augsburg and supplemented with internal clinical data from the University Hospital of Augsburg. The study was approved by the Institutional Review Board of the University Hospital of Augsburg and performed according to the national rules (Approval 25 September 2018—BKF 2018-18). The study has been carried out in compliance with the Helsinki Declaration.

### 2.2. TMA-Construction

In order to allow for high-throughput analysis, selected areas of the tumor tissue of every patient were combined in seven TMAs. Tumor tissue used for TMA construction had been initially fixed in 4% buffered formalin and embedded into paraffin. Patient samples were randomly distributed across TMAs. Replicate tumor cores from each patient were placed one after another. The TMAs contained two cores (1 mm in diameter) of each patient. After microscopic examination of stained H&E slides of the initial six TMAs, there was insufficient tumor tissue, in terms of quantity or quality, for 21 patient samples. Thus, two additional cores of these cases were included in a seventh TMA. In total, 594 tumor cores were included in all TMAs, of which 535 tumor cores had tumor tissue of sufficient quality and 59 had to be excluded from further analysis due to tissue loss during sample preparation or low tumor content. The TMAs were produced manually with the Manual Tissue Arrayer Model MTA-1 (Alpha Metrix Biotech GmbH, Rödermark, Germany). After completing a TMA block, it was heated at 38 °C for 60 min.

### 2.3. MALDI-MSI Measurement

From each TMA, a 4 μm section was adhered to an indium-tin-oxide (ITO) coated glass slide (Bruker Daltonics, Bremen, Germany). Sample preparation has previously been described in detail [21]. Briefly, sample slides were heated to 80 °C prior to dewaxing with xylene (Carl Roth GmbH, Karlsruhe, Germany), and subsequently rehydrated with graded ethanol washes (Carl Roth GmbH). Afterward, samples were subjected to heat-induced antigen retrieval in MilliQ water at 95 °C for 20 min. A trypsin (Promega, Mannheim, Germany) solution was prepared in 40 mM ammonium bicarbonate (Sigma-Aldrich Chemie GmbH, Munich, Germany) to a final concentration of 0.1 µg/µL, for on-tissue digestion. The trypsin solution was sprayed utilizing an automatic sprayer (TM Sprayer, HTX Technologies, Chapel Hill, NC, USA) in 16 cycles with a fixed spraying flow of 150 μL/min (5 × 10^−3^ µg/mm^2^). Sections were subsequently incubated in a humidity chamber at 37 °C for two hours. Following digestion, four cycles of matrix solution (10 mg/mL of α-cyano-4-hydroxycinnamic acid matrix (Sigma-Aldrich Chemie GmbH)) in 70% acetonitrile aqueous solution with 1% trifluoroacetic acid (Carl Roth Gmbh) were sprayed with a defined flow of 120 μL/min (2 × 10^−3^ µg/mm^2^).

MSI was performed using a rapifleX^®^ MALDI Tissuetyper^®^ TOF mass spectrometer (Bruker Daltonics). A peptide calibration standard mix (bradykinin, angiotensin II, angiotensin I, substance P, bombesin, ACTH clip 1–17, ACTH clip 18–39, and somatostatin 28 (Bruker Daltonics)) was used for external calibration. Each spectrum was automatically generated at a spatial resolution of 50 µm using flexControl (Bruker Daltonics) in the mass range of *m*/*z* 600–3200. Five hundred laser shots were acquired for each spectrum at 1 kHz, with a laser power of 70–80%. Laser application was defined as M5 small, beam scan was turned on, with a sample rate of 1.25 GS/s. Real-time smoothing was turned off, while matrix suppression was deflected up to *m*/*z* 479. Global offset attenuator was set at 2%. The measurement regions were defined using flexImaging (Bruker Daltonics). Following MSI measurements, the matrix was removed by two washes in 99.99% methanol (Carl Roth GmbH) for 3 min each, followed by two washings in 99.99% ethanol (Carl Roth GmbH) for 10 s.

### 2.4. Histological Tumor Annotation, Data Processing, and Extraction

TMA sections previously analyzed by MSI were stained with H&E and digitalized utilizing a slide scanner (Aperio CS2, Leica Biosystems, Nussloch, Germany). H&E scans were uploaded, and tumor regions were thoroughly annotated using SCiLS Cloud (discontinued service from Bruker Daltonics). MSI data was processed using SCiLS Lab Pro (Bruker Daltonics) for mass spectrometry and image visualization. Annotations were imported into SCiLS Lab Pro software. Spectra were normalized to the total ion count (TIC).

### 2.5. MS/MS Measurements

For feature identification, cores with the highest concentration of the peptide of interest were traced back to the original block and whole mount tissue sections were used.

MS/MS measurements were performed on a timsTOF fleX mass spectrometer (Bruker Daltonics). The spectra were generated in positive ion mode. Laser power for fragmentation was set at 68% and a beam scan of 95 µm^2^.

Two different tuning methods were used to optimize fragmentation patterns. One tuned for low masses, where the lower masses of the sequence (*m*/*z* < 400) were fragmented with 2000 laser shots and a wider tuning method for fragmentation of higher masses (350 < *m*/*z* < 1303.6) using 10,000 laser shots.

Fragmentation spectra were merged (Figure S1) in Bruker DataAnalysis software (Bruker Daltonics), and an *m*/*z* fragmentation list was generated. Deconvolution of different fragments was performed by MASCOT to search for tentative protein identification, with assignment preference given to *homo sapiens* taxonomy in the SwissProt database [22]. Variable oxidation of the individual amino acids, as well as *N*-terminal acetylation, were taken into consideration. Peptide mass tolerance was defined as ±0.1%, fragment mass tolerance was set at ±0.5 Da, with a maximum of two missed cleavages allowed. Further details about the search output can be consulted in the Supplementary Material (Figures S2 and S3).

### 2.6. Statistical Analysis

Technical reproducibility of MALDI-TOF-MSI has been previously addressed in a few studies [21,23,24,25]. Furthermore, a cross-normalization profile, also succinctly described in Section 2.6.1, was applied to ensure inter-measurement comparability [26,27]. Additionally, cores of liver tissue have been randomly placed in all the TMAs to confirm reproducibility and comparability (Figure S4). As mentioned in Section 2.4, following the MALDI-TOF-MSI measurements and prior to the statistical analysis, the very same sections used for MALDI-MSI measurements were stained with H&E, digitalized, and annotated by a pathologist. Thus, only tumor regions were taken into consideration for statistical analysis.

#### 2.6.1. Supervised Classification

Spectra were preprocessed for intensity profile normalization, re-sampling, spatial denoising, and calculation of a second normalization profile, as previously described [26,27]. Briefly, all spectra were recalibrated to the peptide chemical noise background as previously described in detail [27]. Peptide mass resampling was performed in every data analysis according to the method described by Boskamp et al., where all spectra were resampled to the expected *m*/*z* values according to the peptide mass rule with a width of the bins of 0.4 Da in the *m*/*z* range from 700 Da to 2000 Da [26]. For the TIC normalization, all spectra were divided by the sum of their respective intensities. Intensity profile normalization was carried out as described by Boskamp et al., where the intensities of the spectra are transformed so that their distribution matches a reference profile [26]. The reference profile was always calculated based on the spectra of SOP-compliant datasets. The mass axis was partitioned into two equally sized intervals for the normalization. For spatial denoising, the intensities for all *m*/*z* values were spatially smoothed using a gaussian kernel inside a radius of 105 µm with a sigma of 75 µm. The peak picking was performed on the global skyline spectrum in SCiLS Lab (Bruker Daltonics) with a peak width of 0.4 Da and peak aggregation mode “sum”. The monoisotopic mass and the second isotopic mass of the most intense (signal-to-noise ratio greater than 3 units) features in the *m*/*z* range 600–2000 were selected. Afterward, spectra of individual spots were exported to .csv-format and imported to R statistical software (version 3.6.3) and RStudio 1.2.5033.

The dataset was split into training (70%) and test (30%) set, with method control set to 10-fold cross-validation for all models. Classification models were fitted using the “caret” package on R [28]. Linear discriminant analysis (LDA) was fitted using the method “lda”. Random Forest (RF) was fitted using the method “ranger”, with the number of trees set to 500. Tuning parameter ‘min.node.size’ was held constant at a value of 1. Accuracy was used to select the optimal model using the largest value. The final values used for the model were mtry = 131, splitrule = gini and min.node.size = 1. K-Nearest Neighbors (kNN) was fitted using the method “knn”. Accuracy was used to select the optimal model using the largest value. The final value used for the model was *k* = 5. Support vector machine (SVM) was fitted using the method “svmRadialWeights”. Tuning parameter ‘sigma’ was held constant at a value of 0.006480179. Accuracy was used to select the optimal model using the largest value. The final values used for the model were C = 1 and Weight = 1.

The fitted models were used to predict the test data subset. The accuracy, sensitivity, and specificity were calculated based on the results of the confusion matrix (Table S1).

#### 2.6.2. Unsupervised Analysis

Without further preprocessing, normalized data was exported as a .csv file and imported to R statistical software (Rstudio 3.5.1). Prior to statistical analysis, a mean spectrum was created per patient. The partitioning around medoids method (PAM) was used to cluster the mean intensities of each *m*/*z* value into three groups: high intensity, medium intensity, and low intensity. By employing a three tier-partitioning, we are separating the high intensity from the medium intensity features, which in a two-tiered system would have been forced in either the high-intensity or the low-intensity classes. By adding a third tier, we accommodate enough complexity, but at the same time, we decrease the number of potential features that carry on to the next step of the analysis. For clustering, the package “cluster” for R, using the method “pam”, was adopted [29].

The patient population was divided into exploration and test sets; patients with an odd number were included in the exploration set (*n* = 138), and patients with an even study number were included in the test set (*n* = 138). Kaplan–Meier (KM) analyses were performed on a manually created peaklist (1007 *m*/*z* features) according to the occurrence of distant metastasis. 13 patients were excluded from KM analysis because information about the time-point they developed distant metastases was not available. The initial univariate event analysis in the exploration and test sets was performed using the “survival” package for R employing the “KM” method (log-rank test) [30]. For prognostic KM analysis, a two-stage approach was used in order to reduce the false positive and negative results due to multiple testing (KM analysis of 1007 *m*/*z* values). In this context, a result was only considered statistically significant if *p* was <0.05 in the test set. The Statistical Package for the Social Sciences (SPSS, Chicago, IL, USA, version 24.0) was used for comparison of clinicopathological data between exploration and test set. Pearson’s chi-squared test was used to evaluate nominal data. Mann–Whitney *U* test was applied for comparisons between continuous and ordinal variables between two groups. KM analyses of selected features were also re-performed with SPSS.

#### 2.6.3. Immunohistochemical Analysis

For validation, immunohistochemical stainings were performed using serial sections from the same TMAs utilized for MALDI-MSI analysis. The following antibodies were investigated: cytokeratin 15 (antibody: BioSB, Bioscience For The World, monoclonal rabbit antibody; dilution 1:100; DAB OptiView IHC Detection Kit; immunostainer, Roche Benchmark Ultra) and collagen type III alpha-1 chain (antibodies-online.com, ABIN303354, polyclonal rabbit antibody; dilution 1:50; DAB Opti View IHC Detection Kit; immunostainer, Roche Benchmark Ultra). The staining results were evaluated by a pathologist and classified in a three-tiered grading system (negative, weak positive, and strong positive) to enable comparability.

## 3. Results

### 3.1. Patient Characteristics

The patient characteristics are summarized in Table 1. Apart from the histological subtype, there are no significant differences between the exploration and test set. In the exploration set, the number of carcinomas not otherwise specified (NOS) was lower in contrast to the test set (*p* = 0.019).

### 3.2. Prediction of Metastasis

#### 3.2.1. Supervised Classification

To better account for intra-tumor heterogeneity, supervised classification was performed on all individual spectra (271,719) from the histologically annotated tumor regions. Seventy percent of the spectra were used for the training and validation of four classification models, while the remaining 30% of the spectra were subsequently utilized to test the classification models. Table 2 summarizes the classification results.

#### 3.2.2. Unsupervised Classification

KM analysis of the 1007 selected *m*/*z* values revealed 39 features with a *p*-value < 0.05 in the exploration set (Table S2). In the test set, seven features showed a *p*-value < 0.1, and two features showed a *p*-value < 0.05 (Table 3). The prognostic impact of *m*/*z* = 656.25 was not taken into consideration since the results of KM analysis from the exploration and test sets were not consistent.

The intensity thresholds and cluster sizes for the features *m*/*z* 1821.8 and *m*/*z* 1303.6 are outlined in Table 3.

### 3.3. m/z 1821.8

**Figure 1:**
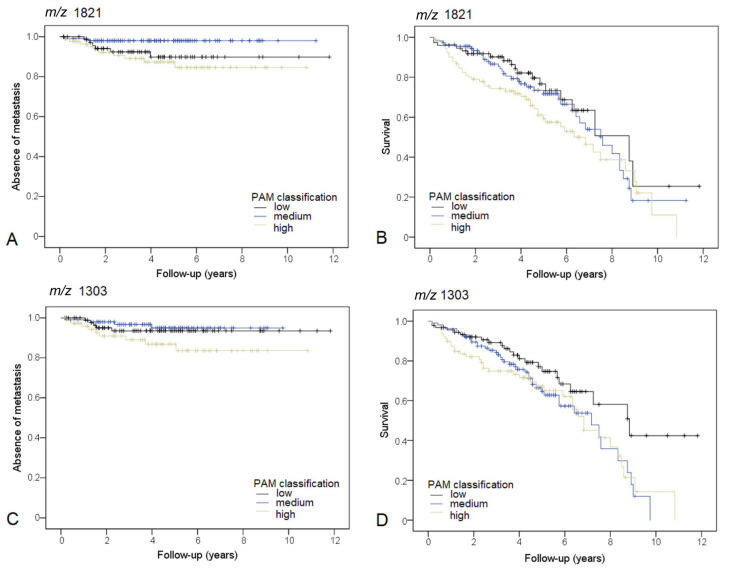
(**A**) KM curve for the occurrence of distant metastasis. Patients are stratified based on PAM classification of *m*/*z* 1821.83. (**B**) KM curve for patient survival. Patients are stratified based on PAM classification of *m*/*z* 1821.83. (**C**) KM curve for the occurrence of distant metastasis. Patients are stratified based on PAM classification of *m*/*z* 1303.06. (**D**) KM curve for overall survival. Patients are stratified based on PAM classification of *m*/*z* 1303.06.

The three groups, defined by the mean intensities of *m*/*z* 1821.8, differed significantly concerning the occurrence of distant metastasis in the exploration (*p* = 0.007) and test (*p* = 0.01) sets. A high intensity of *m*/*z* 1821.8 was associated with metastasis in comparison to the medium intensity group in the exploration (*p* = 0.036) and test (*p* = 0.03) set, respectively. Figure 1A depicts the KM curve for the complete study cohort (*p* = 0.026). Furthermore, high intensity of *m*/*z* 1821.8 was significantly associated with shorter overall survival in comparison to low intensity (*p* = 0.037, *n* = 276). For reference, colon cancer-specific survival can be observed in Figure S5.

### 3.4. m/z 1303.6

The three groups, defined by the mean intensity of *m*/*z* 1303.6, differed significantly concerning the occurrence of distant metastasis in the exploration set (*p* = 0.041), with the worst prognosis for the high-intensity group. The KM analysis did show a trend in the test set (*p* = 0.091). Pairwise comparison between the medium and high-intensity group showed a *p*-value of 0.056 in the test set. A KM curve for the complete study cohort is shown in Figure 1C (high intensity versus medium intensity *p* = 0.037, high intensity versus low-intensity *p* = 0.134). High intensity, as well as medium intensity (*p* = 0.042 and *p* = 0.020, respectively) of *m*/*z* 1303.6, were significantly associated with a shorter overall survival in comparison to low intensity in the study population.

*m*/*z* 1303.6 and *m*/*z* 1821.8 were both considered relevant and tentative MS/MS identification was carried out. *m*/*z* 1821.8 did not give a clear fragmentation spectrum. *m*/*z* 1303.6 fragmentation spectrum (Figure S1) was used for protein identification. The mass list was used for MASCOT search (Figures S2 and S3), which yielded one hit for *m*/*z* 1303.6: collagen type-III alpha-1 (Col3A1).

## 4. Discussion

In this study, we analyzed samples from 276 patients with UICC stage I/II colon cancer to investigate the prognostic capability of proteomic analysis by MALDI-MSI.

To address this challenging issue, we have approached the data analysis in two different ways: a supervised and an unsupervised approach. For the supervised approach, four different machine learning classification algorithms were fitted, validated, and tested. The classification was performed using the 130 most intense features within the measurement range. Results from the supervised classification are promising (Table 2) with accuracy values over 90% for SVM, kNN, and RF. LDA is the least accurate model from the ones employed in this study, achieving only 57% accuracy. All classification methods also presented satisfying sensitivity, making this approach a promising option for the prediction of colon cancer progression. Nonetheless, due consideration should be given to the results, since the classes are not balanced: approximately 90% of included patients did not develop metastasis during follow-up. Despite being a realistic approximation for the actual numbers in the overall population, the accuracy results also reflect said unbalance, which is denoted by a lower classification specificity. Furthermore, similarities between the two classes are evidenced by the principal component analysis (PCA, Figure S6). Feature selection did not yield any meaningful values (we considered meaningful any AUROC value over 0.75, Table S4), however, a couple of features reoccurred as prominent in our models (Table S5), as obtained by calculation of forward feature extraction, namely *m*/*z* 886.4 (which has been identified as a collagen-1; Figure S7), *m*/*z* 781.4 (which we did not succeed in identifying), and *m*/*z* 1032.5 (which has been identified as Histone H3; Figure S8).

The second approach was based on unsupervised statistical analysis followed by prognostic prediction, where 1007 *m*/*z* features were individually evaluated for their potential correlation with worse patient outcomes. Two features of particular interest were identified: *m*/*z* 1821.8 and *m*/*z* 1303.6.

*m*/*z* 1821.8 was a significant predictor of the occurrence of metastasis in the exploration and the test set (*p* < 0.05). Furthermore, the high-intensity group of *m*/*z* 1821.8 had a shorter overall survival in comparison to the low-intensity group (*n* = 276, *p* = 0.037). We have performed MS/MS for tentative identification of this peptide. However, it was not possible to get a good fragmentation spectrum due to the low abundance of the *m*/*z* 1821.8 peptide ion, and due to the presence of another peptide ion with an analog mass-to-charge ratio. In addition, a peptide ion at *m*/*z* 1820.8 was present in the tissue samples with higher spectral intensity, making the isolation of the intended parent ion even more challenging. Based on a literature search, we found that *m*/*z* 1821.8 was linked to CK 15 [31]. CK 15 has been identified as a marker of stem cells in the epidermis and cornea [32,33,34,35] and has been described as a biomarker of progression in esophageal carcinomas [36]. Nevertheless, the expression of CK 15 could not be confirmed by immunohistochemistry utilizing our patient cohort (Figure S9). This could be due to low levels of protein expression within the tissue sections. Moreover, not to be disregarded, *m*/*z* 1821.8 could be linked to another protein in colon cancer.

*m*/*z* 1303.6 was also found to be a predictor of metastasis in the exploration set (*p* < 0.05). Pairwise comparison between the medium and high-intensity groups showed a *p*-value of 0.056 in the test set. Furthermore, high intensity, as well as medium intensity of *m*/*z* 1303.6 were significantly associated with shorter overall survival in comparison to low intensity (*p* = 0.042 and *p* = 0.020, respectively) (*n* = 276). *m*/*z* 1303.6 was identified as collagen alpha-1 (III) chain (Col3A1) by MS/MS analysis. Collagen is primarily a part of the stroma, being the main constituent of the extracellular matrix surrounding solid tumors. It has been shown that collagen cross-linking promotes tumor cell invasion, stromal activation, and desmoplasia [37]. Especially in colon carcinoma, tumor-stroma proportion and its prognostic significance have come into the scientific focus in recent years [38,39,40]. However, the epithelial regions were thoroughly annotated by a pathologist, hence only the tumor content was evaluated. Therefore, we can with certainty attribute the prognostic significance of *m*/*z* 1303.6/Col3A1 to the expression in epithelial tumor cells. This seems counterintuitive, but in a previous study it has been shown that epithelial but not stromal expression of Col3A1 is a prognostic indicator of colorectal carcinoma [41]. Kehlet et al. have also established the relationship between collagen, namely the formation of type-III collagen, and colorectal cancer invasiveness and metastatic lesions in stage IV patients [42]. Our results corroborate these findings and give the indication that there might be a significant increase in the collagen content in more aggressive colorectal carcinomas, that tend to develop metastasis. Further confirmation of the results was attempted by immunohistochemical staining for Col3A1 utilizing serial sections of all TMAs. There was only a small correlation (Pearson’s correlation coefficient: 0.17) within the immunohistochemistry results, which did not allow further confirmation (Table S6 and Figure S10). As MALDI-MSI is a more sensitive technique than immunohistochemistry, differences in the collagen levels might not be strong enough to be noticeable by immunohistochemistry, in particular since the evaluation was not performed utilizing a digital image analysis program. Furthermore, the molecule found in situ underwent hydroxylation, which might also be the reason why the employed immunohistochemical staining did not show more marked differences. Moreover, *m*/*z* 862.4, identified by the supervised machine learning approaches, is also a fragment of collagen, further supporting our findings. Nonetheless, an additional MSI evaluation of the tissue digested with collagenase should be considered and might give a better understanding of the collagen contents and changes in colon cancer.

The quantitative prognostic value of Histone H3 (*m*/*z* 1032.5) was not determined in this study as the AUROC values were low. Moreover, when interpreting the study results, it must be considered that patients with a relatively short follow-up (over two months) were also included in the study. As a result, some of the patients could have developed metastasis later on, which was not known at the time of this study. However, in the Kaplan–Meier analysis, the observation period is taken into account, and to keep the sample number meaningful, we had to compromise on a shorter follow-up time.

It is nearly impossible to compare both data analysis approaches. On the one hand, supervised classification provides a more accurate classification based on the given input, which is the trend of the most intense features in the annotated tumor tissue, but it can also overlook less intense peaks (that are not included in the classification to avoid overfitting the data). On the other hand, the supervised approach uses a combination of features for the classification and relies on a set of significant features that together contribute to the classification, thus making it difficult to single out one specific *m*/*z* value as the most prominent for classification. In our supervised approach, classification was carried out on individual measurement spots (50 µm measurement pitch), which better accounts for intra-tumor heterogeneity. For the unsupervised approach, clustering was carried out using the average spectra of the tumor region of each patient. As clustering does not rely on the input information for the data segmentation, it can be utilized to better detect smaller differences in a larger pool of features, without the risk of overfitting the data. As we see it, the presented approaches can be complementary and work synergistically to provide information about colorectal tumor progression.

Up until now, limited information on prognostic biomolecules of UICC stage I/II colon cancer on FFPE tissue has been gathered by MSI. Meding et al. have identified metabolites, peptides/proteins correlating with lymph node metastasis in fresh frozen sections of colon cancer [43]. Hinsch et al. have also applied MALDI-MSI to FFPE tissue and found eight features associated with poor clinical outcome [44]. One of these relevant features identified in their study was *m*/*z* 1477, which has been identified as collagen α-2, further supporting our hypothesis that collagen plays a relevant role in the progression of colon cancer. A recent study by Boyaval et al., using MALDI-MSI has reported a correlation between poor outcomes of patients diagnosed with stage II colorectal cancer and the spreading of the cancer signature glycosylation into the adjacent stroma at the interface [45]. Other efforts to determine tumor prognosis have also been carried out by utilizing other MSI techniques. Two other studies have shown that rapid evaporative ionization mass spectrometry (REIMS) can be used not only to differentiate normal mucosa from adenoma and colorectal cancer but can also aid in tumor characterization and prediction of lymph node micrometastases [46,47]. Veselkov and co-workers have also resourced desorption electrospray ionization (DESI) MSI for the characterization of the lipid profiles of the colorectal cancer tumor microenvironment [48].

Despite employing different approaches (MALDI, DESI, or REIMS), it seems that mass spectrometry imaging might have the necessary answers for a more complete colorectal tumor profiling and prognostic prediction. From our analysis, we show that MALDI-MSI is capable of accurately classify colon cancer according to the tumor progression status. We also advance a couple of features that may correspond to significant prognostic markers for UICC stage I/II colon cancer metastasis. Identification of new biomarkers that reflect changes in the tumor microenvironment could give a much needed indication about tumor prognosis. Additionally, they could provide a viable target for blocking tumor invasion, stopping tumor progression, novel treatment approaches, and thus improving patient outcomes in colon cancer.

## 5. Conclusions

Currently, no sufficient biomarkers exist to delineate patients with UICC stage I/II colon cancer that would benefit from adjuvant therapy from those who would not benefit. Utilizing MALDI-MSI, a highly sensitive technique that can detect proteomic changes within intact tissue sections in a spatially resolved manner, we were able to establish accurate classification models that could predict the occurrence of distant metastasis in 276 patients. Furthermore, two *m*/*z* values (*m*/*z* 1303.6 (Col3A1) and *m*/*z* 1821.8) could be identified that show encouraging results for the identification of new prognostic biomarkers in colon cancer. This approach highlights the possibilities of combining histology with MALDI-MSI and sophisticated bioinformatics analysis. Moving forward, validation of these promising findings in an independent patient cohort as well as further investigations into the role of collagens in colon cancer progression could lead to a better delineation in the patient treatment and a more profound understanding of colorectal cancer progression.

## Figures and Tables

**Table 1:** Overview of patients’ characteristics.

Characteristics	All Patients		Exploration Set (*n* = 138)		Test Set (*n* = 138)		*p*-Value
	*n*	%	*n*	%	*n*	%	
Age (years ± SD)	69.9 ± 10.4		69.9 ± 10.9		70.1 ± 10.0		NS
Follow-up (years ± SD)	4.2 ± 2.39		4.0 ± 2.4		4.4 ± 2.4		NS
Sex							NS
Female	114	41.3%	54	39.1%	60	43.5%	
Male	162	58.7%	84	60.9%	78	56.5%	
UICC							NS
I	112 (40.5%)	40.6%	54	39.1%	58	42.0%	
II	164 (59.4%)	59.4%	84	60.9%	80	58.0%	
pT							NS
1	12	4.3%	6	4.3%	6	4.3%	
2	100	36.2%	48	34.8%	52	37.7%	
3	155	56.2%	80	58.0%	75	54.3%	
4	9	3.3%	4	2.9%	5	3.6%	
Grading							NS
Low	218	79.0%	109	79.0%	109	79.0%	
High	58	21.0%	29	21.0%	29	21.0%	
Differentiation							0.019
Adenocarcinoma, NOS	241	87.3%	114	82.6%	127	92.0%	
Specific Subtype	35	12.7%	24	17.4%	11	8.0%	
Death							
All causes	102	37.0%	48	34.8%	54	39.1%	NS
Colon Cancer specific	26	9.4%	11	8.0%	15	10.9%	NS
Distant Metastasis							NS
No	245	88.8%	126	91.3%	119	86.2%	
Yes	31	11.2%	12	8.7%	19	13.8%	

UICC = Union Internationale Contre le Cancer, SD = standard deviation, NOS = no special type, NS = not significant.

**Table 2:** Summary of the supervised classification results of the test set. 130 *m*/*z* values (*m*/*z* range 600–2000) with 10-fold cross-validation. RF = random forest (*n* = number of trees), kNN = k-nearest neighbors, SVM = support vector machine, LDA = linear discriminant analysis.

Supervised Classification Results	RF (*n* = 500)	kNN	SVM	LDA
Balanced accuracy	0.9731	0.9828	0.9217	0.5700
Sensitivity	0.9991	0.9963	0.9992	0.9887
Specificity	0.9471	0.9693	0.8442	0.1513

**Table 3:** Overview of clustered mean intensities with PAM of *m*/*z* 1821.8 and *m*/*z* 1303.6.

*m*/*z* Features	Low Intensity	Medium Intensity	High Intensity
***m*/*z* 1821.8**			
*n* = 276	27%	40%	33%
Mean intensity	1.23	1.77	2.39
Intensity range	0.790–1.526	1.532–2.015	2.016–6.80
***m*/*z* 1303.6**			
*n* = 276	33%	38%	29%
Mean intensity	5.73	9.16	13.78
Intensity range	1.58–7.52	9.16–10.84	11.17–21.74

PAM = partitioning around medoids.

## Data Availability

The data presented in this study are available on request from the corresponding author.

## Supplementary Materials

The following are available online at www.mdpi.com/2072-6694/13/21/5371/s1. Figure S1: Average spectra from liver core in the TMAs used as reference for assessment of inter-measurement comparability, Table S1: Confusion matrix results for the four models, Table S2: Summary of all *m*/*z*-values with *p* < 0.05 in the exploration set, Figure S2: KM curve for colon-cancer-specific survival, Figure S3: Fragmentation spectra of the *m*/*z* 1303.6, Figure S4: MASCOT score histogram, Figure S5: MASCOT sequence match view, Figure S6: Plot of the PCA analysis based on the 130 most intense *m*/*z* features, Table S3: Peptide identification by MS/MS, Figure S7: Tentative MS/MS identification of *m*/*z* 886.4, Figure S8: Tentative MS/MS identification of the *m*/*z* 1032.5, Figure S9: Immunohistochemistry of cytokeratin 15, Figure S10: Immunohistochemistry of Col3A1, Table S4: Relationship between *m*/*z* 1303 PAM classification and Col3A1 immunohistochemistry, Table S5: Pairwise comparison of the Area Under Curve of the Receiver Operator Characteristic, Table S6: Most prominent features resulting from forward feature calculation.
