# Supplementary material for: A Mass Spectrometry Imaging Based Approach for Prognosis Prediction in UICC Stage I/II Colon Cancer
Source: Cancers (Basel). 2021 Oct 26;13(21):5371. doi: 10.3390/cancers13215371 (PMC8582467; doi:10.3390/cancers13215371)
Supplement: Supplementary file 1 [file cancers-13-05371-s001.zip › cancers-1384840-supplementary.pdf]

Article

A Mass Spectrometry Imaging Based Approach for Prognosis Prediction in UICC Stage I/II Colon Cancer

Benedikt Martin [1,†], Juliana P. L. Gonçalves [2,†], Christine Bollwein [2], Florian Sommer [3], Gerhard Schenkirsch [4], Anne Jacob [2], Armin Seibert [5], Wilko Weichert [2], Bruno Märkl [1] and Kristina Schwamborn [2,*]

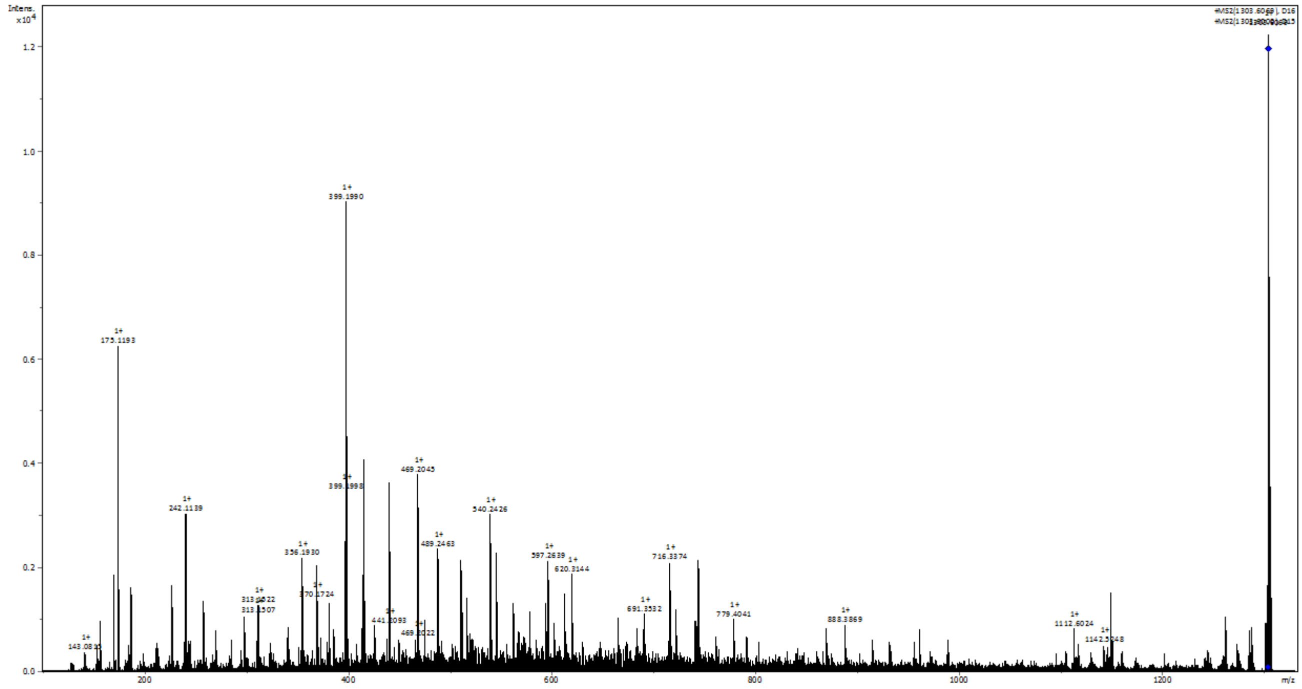

Figure S1. Fragmentation spectrum (MS/MS, timsTOF fleX) of the *m/z* 1303.6. These spectra resulted of a defined isolation mass of *m/z* 1303.60 (isolation width of 5.00 Da). These spectra are a reconstitution of two different spectra: One spectrum was recorded using a tuning method for lower masses (with fragmentation *m/z* < 400) and a second spectrum was recorded with a method for a wider range of masses (*m/z* > 350).

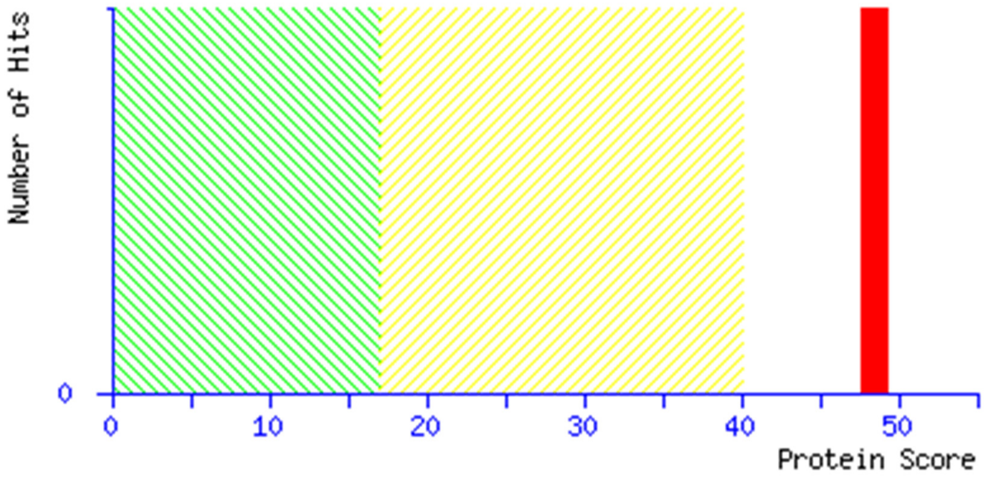

Figure S2. Mascot Score Histogram.

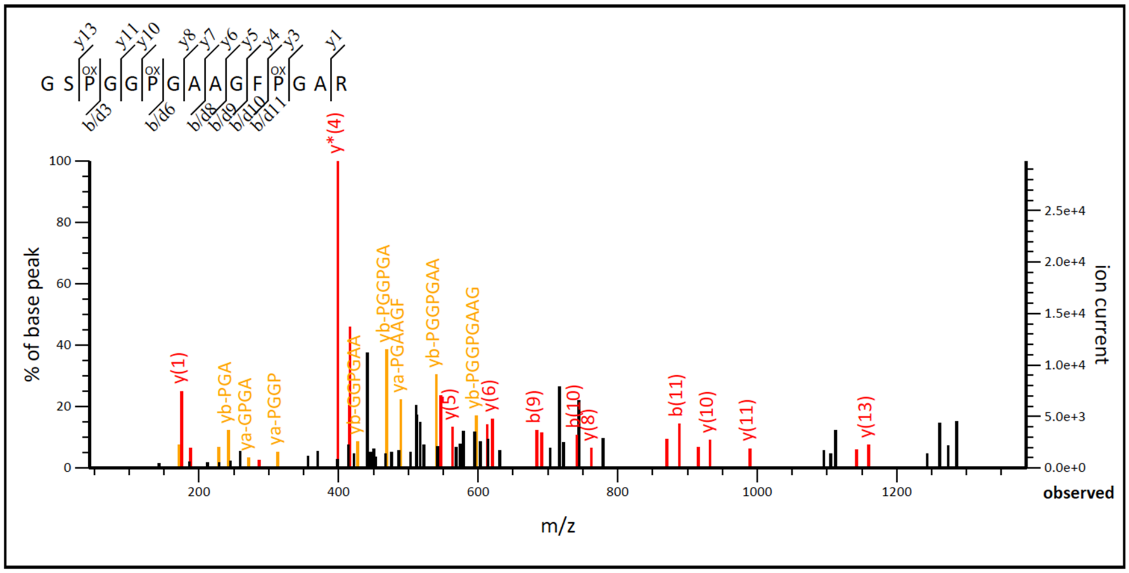

Figure S3. MASCOT Sequence Match View.

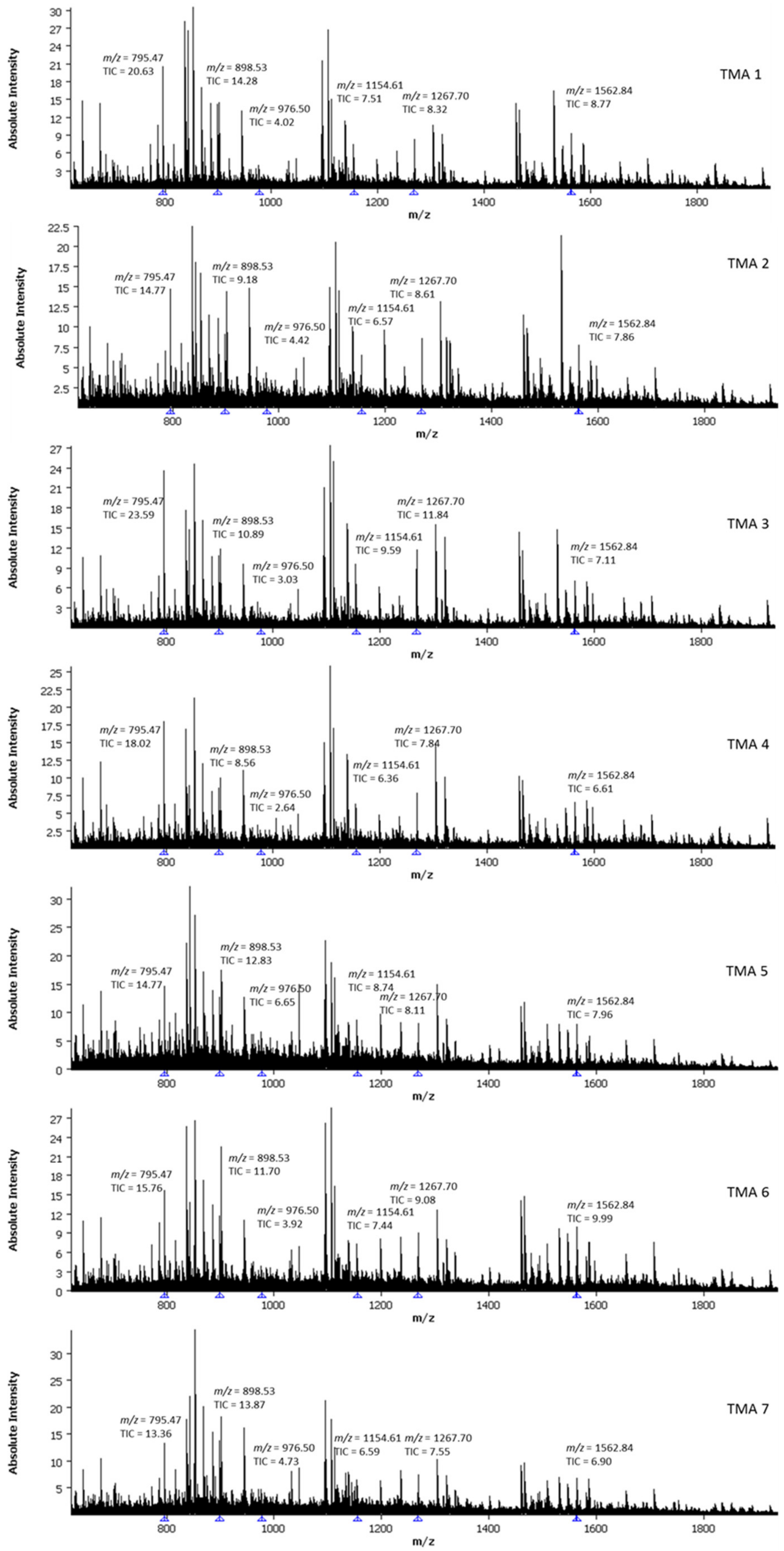

Figure S4. Average spectra from liver cores in the TMAs used as reference for assessment of inter-measurement comparability.

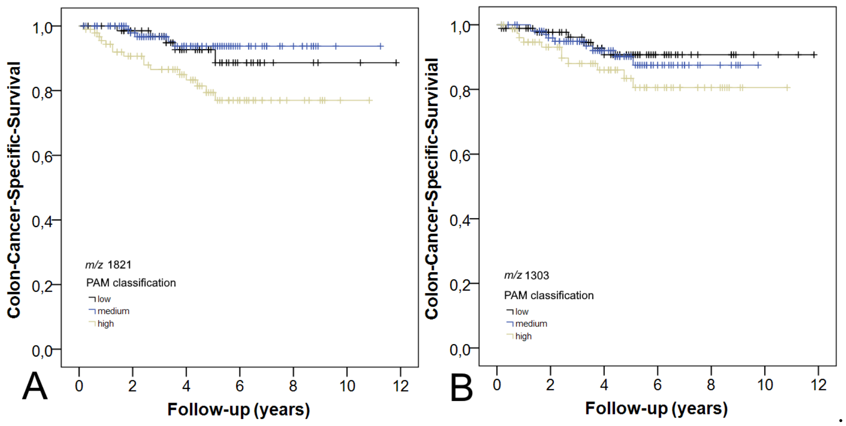

Figure S5. A) KM curve for colon-cancer-specific survival. Patients are stratified based on PAM classification of *m*/*z* 1821.83 (p = 0.008; medium versus high p = 0.005; low versus high p = 0.052; medium versus low p = 0.55; n = 276). B) KM curve for colon-cancer-specific survival. Patients are stratified based on PAM classification of *m*/*z* 1303.6 (p = 0.24; medium versus high p = 0.21; low versus high p = 0.12; medium versus low p = 0.70; n = 276).

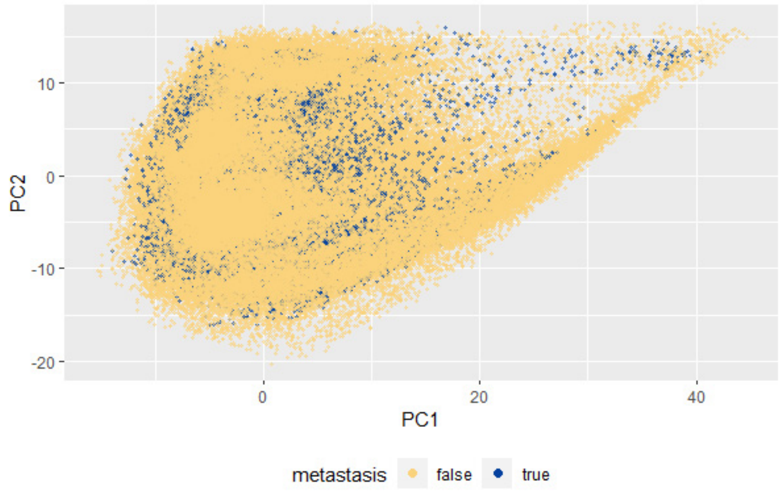

Figure S6. Plot of the PCA analysis based on the 130 most intense *m*/*z* features. Patients that developed metastasis are represented in blue, and the patients that did not develop metastasis during follow-up are represented in yellow.

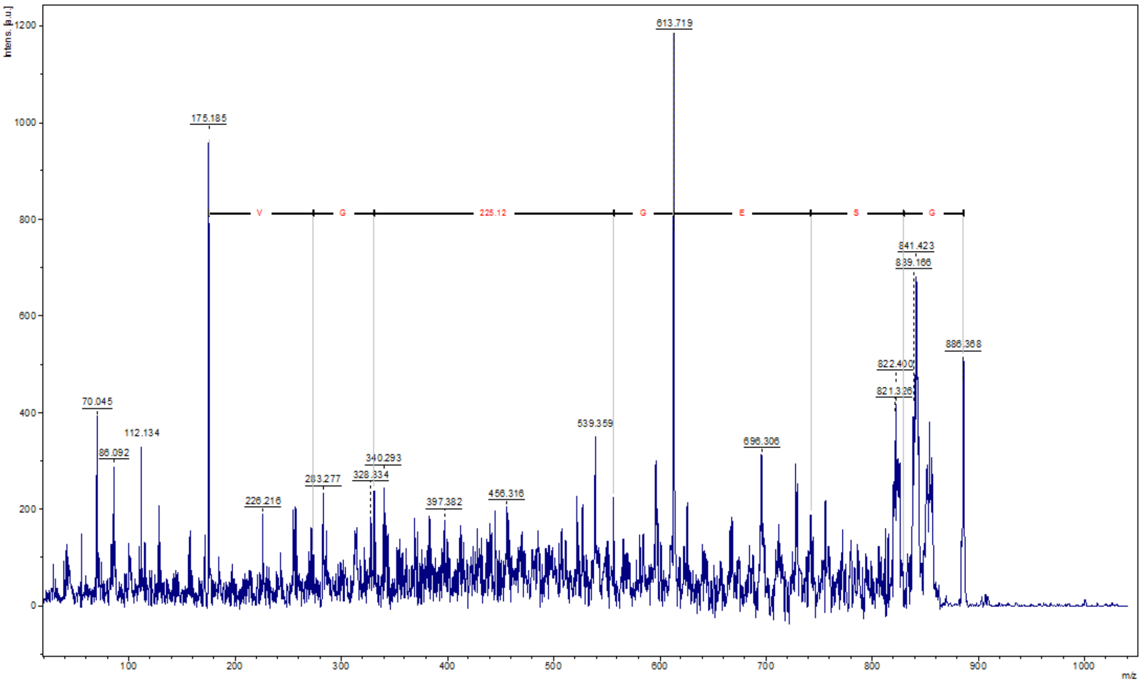

Figure S7. Tentative MS/MS identification of *m*/*z* 886.4**.**

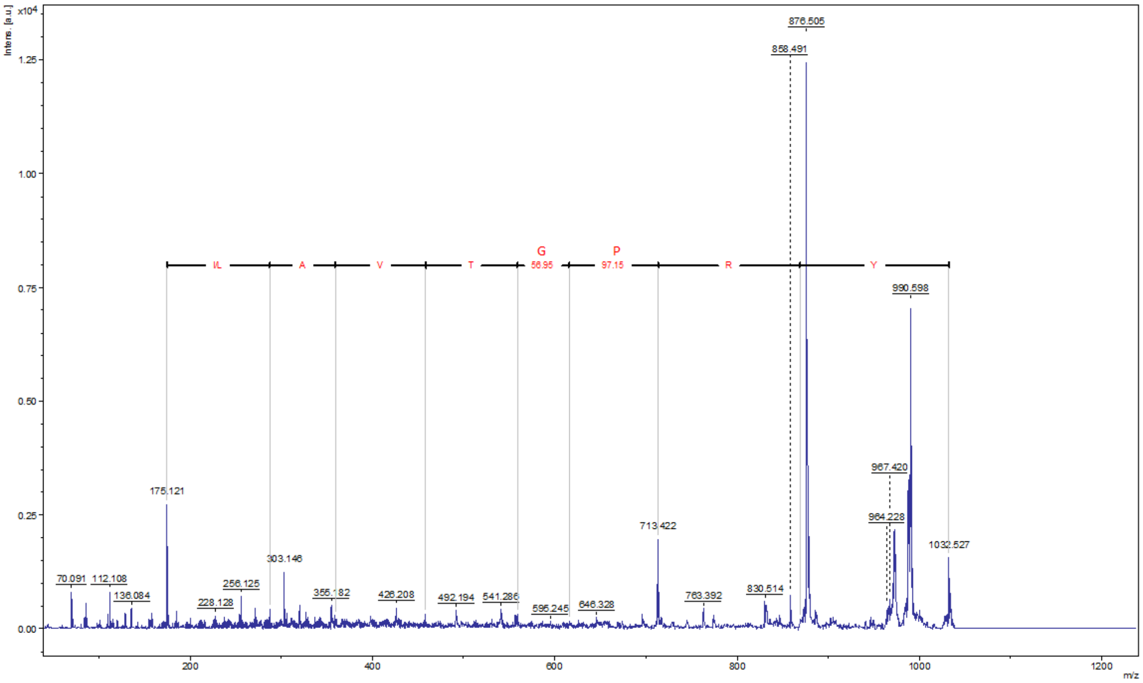

Figure S8. Tentative MS/MS identification of the *m*/*z* 1032.5**.**

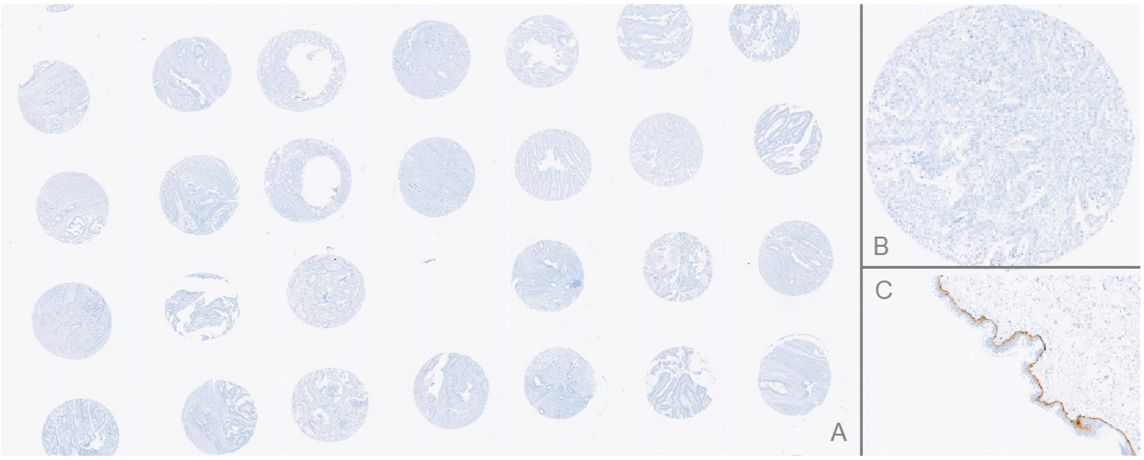

Figure S9. Immunohistochemistry of cytokeratin 15; A) section of one representative TMA; B) negative staining result of one core in detail; C) positive on slide control**.**

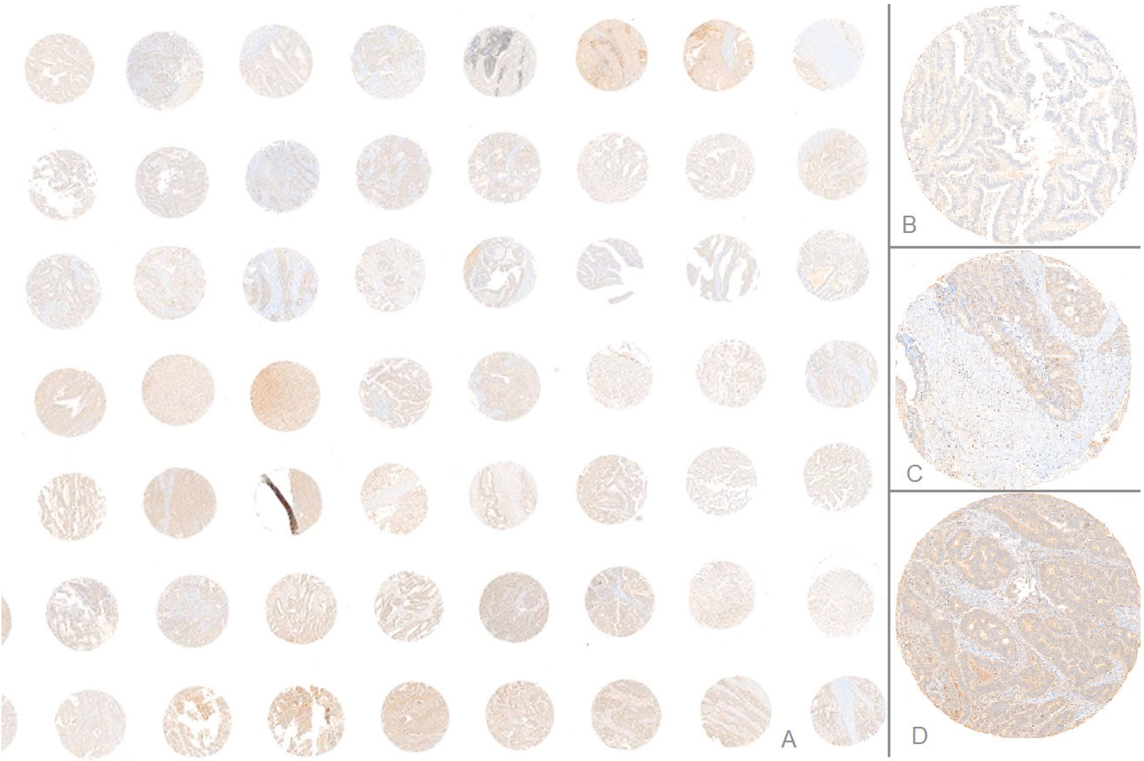

Figure S10. Immunohistochemistry of Col3A1; A) section of one representative TMA; B) negative staining result; C) weak positive; D) strong positive**.**

Table S1. Confusion matrix results for the four models. RF – random forest, kNN – k-nearest neighbor, SVM – support vector machine, LDA – linear discriminant analysis.

	RF (500 trees)	kNN	SVM	LDA
**Accuracy**	0.9937	0.9935	0.9831	0.902
**95% CI**	(0.9931, 0.9942)	(0.9929, 0.994)	(0.9822, 0.984)	(0.8999, 0.904)
**No Information Rate**	0.8965	0.8965	0.8965	0.8965
**P-Value [Acc > NIR]**	< 2.2e-16	<2e-16	< 2.2e-16	9.534e-08
**Kappa**	0.9653	0.9649	0.9026	0.2096
**Mcnemar's Test P-Value**	< 2.2e-16	0.573	< 2.2e-16	< 2.2e-16
**Sensitivity**	0.9991	0.9963	0.9992	0.9887
**Specificity**	0.9471	0.9693	0.8442	0.1513
**Pos Pred Value**	0.9939	0.9965	0.9823	0.9098
**Neg Pred Value**	0.9914	0.9677	0.9914	0.6069
**Prevalence**	0.8965	0.8965	0.8965	0.8965
**Detection Rate**	0.8956	0.8931	0.8957	0.8863
**Detection Prevalence**	0.9011	0.8963	0.9119	0.9742
**Balanced Accuracy**	0.9731	0.9828	0.9217	0.5700

Table S2. Summary of all *m*/*z*-values with $p < 0.05$ in the exploration set. Highlighted in green are features with $p < 0.05$ in the exploration and test set; highlighted in blue are features with $p < 0.1$ in the test set.

Overview of p-values of the Kaplan-Meier analysis*		
	**Exploration set**	**Test set**
m/*z*	**p-value**	**p-value**
603.32	0.000225757	0.40131093
1820.78	0.00117734	0.08585357
623.33	0.002198562	0.87550545
1501.75	0.002811342	0.51215044
666.34	0.003393999	0.73439213
1821.83	0.0075557	0.01281608
616.39	0.010682376	0.05085195
710.39	0.011656222	0.42320477
1998.01	0.014561757	0.53860067
1176.55	0.016260784	0.85765962
1598.79	0.016290998	0.57955638
920.47	0.017738762	0.74103349
1288.67	0.026648082	0.51482455
656.26	0.028644741	0.01452846
759.41	0.030524084	0.66207935
1133.57	0.031270459	0.0613477
1150.59	0.031808426	0.80806958
602.32	0.034813685	0.70019195
607.21	0.035259135	0.49593758
1039.55	0.035365338	0.46934992
2951.38	0.035938029	0.42166608
1622.73	0.036206937	0.24862981
613.32	0.036874602	0.89095681
1094.56	0.037463774	0.68328973
2706.13	0.038607591	0.06018686
1076.57	0.039304063	0.26619165
1303.6	0.040716569	0.09142353
2712.17	0.040834712	0.36546008
2953.4	0.041407977	0.52075282

647.34	0.041438481	0.17819513
1321.67	0.042150831	0.1043217
673.4	0.042281203	0.75931796
2104.09	0.043208088	0.16692916
2295.03	0.043521947	0.10900229
1530.75	0.044513376	0.92311936
2334.14	0.045759626	0.27342927
620.31	0.0467169	0.25932014
2369.98	0.049334071	0.51179223
974.53	0.049571296	0.56685499

Table S3. Peptide identification by MS/MS.

**Measured *m/z.***	**Sequence**	**Description**
1303.6	R.GSPGGPGAAGFPGAR.G + 3 Oxidation (P)	CO3A1 Collagen alpha-1 (III) chain
1032.5	R.YRPGTVALR.E	Histone H3
886.4	R.GSEGPQGVR.G	CO1A1 Collagen alpha (I) chain

Table S4. Pairwise comparison of the Area Under Curve of the Receiver Operator Characteristic (AUC_ROC). Possible identification based on previously published reports.

m/z	**AUC_ROC**		**Possible ID**
1138.5896	0.5873484	+1	Collagen alpha-1(III) chain [1]
943.581177	0.5766103	+1	
944.555237	0.572499	+1	Histone 2A[1]
1198.68958	0.5701885	+1	Actin[1]
1111.60791	0.556772	+1	Annexin A2[1]
1320.69153	0.5543657	+1	Keratin, type II cytoskeletal[1]
960.481262	0.5542658	+1	
805.409668	0.5536868	+1	
958.533142	0.5526416	+1	Stress-70 protein, mitochondrial [2]
971.585632	0.5496143	+1	

Table S5. Most prominent features resulting from forward feature calculation**.**

m/z	**Accuracy**
781.3989	0.8982438
886.4749	0.8993368
1032.585	0.8983325

Table S6. Relationship between *m/z* 1303 PAM classification and Col3A1 immunohistochemistry. Pearson's correlation coefficient: 0.17.

**PAM clustering of *m/z* 1303**	**Immunohistochemistry Col3A1**					
	**negative**		**weak**		**strong**	
	n	%	n	%	n	%
low	28	32	36	41	23	26
medium	29	28	54	53	19	19
high	33	43	35	46	8	11

References:

1. Groseclose, M.R.; Massion, P.P.; Chaurand, P.; Caprioli, R.M. High-throughput proteomic analysis of formalin-fixed paraffin-embedded tissue microarrays using MALDI imaging mass spectrometry. *Proteomics* **2008**, *8*, 3715–3724,

doi:10.1002/pmic.200800495.

2. Casadonte, R.; Kriegsmann, M.; Kriegsmann, K.; Hauk, I.; Meliß, R.R.; Müller, C.S.L.; Kriegsmann, J. Imaging Mass Spectrometry-Based Proteomic Analysis to Differentiate Melanocytic Nevi and Malignant Melanoma. *Cancers 2021, Vol. 13, Page 3197* **2021**, *13*, 3197, doi:10.3390/CANCERS13133197.
